# Multiple Infection and Microdiversity among *Helicobacter pylori* Isolates in a Single Host in India

**DOI:** 10.1371/journal.pone.0043370

**Published:** 2012-08-27

**Authors:** Rajashree Patra, Santanu Chattopadhyay, Ronita De, Prachetash Ghosh, Mou Ganguly, Abhijit Chowdhury, T. Ramamurthy, G. B. Nair, Asish K. Mukhopadhyay

**Affiliations:** 1 National Institute of Cholera and Enteric Diseases, Kolkata, India; 2 Centre for Liver Research, School of Digestive and Liver Diseases, Institute of Post Graduate Medical Education & Research, Kolkata, India; Veterans Affairs Medical Center (111D), United States of America

## Abstract

*Helicobacter pylori* is one of the most diverse bacterial species that chronically infects more than 70% of Indian population. Interestingly, data showing microdiversity of the *H. pylori* strains within a particular gastric niche remained scarce. To understand the extent of genetic diversity among *H. pylori* strains within a given host, 30 patients with gastro-duodenal problems were subjected to endoscopy and from each patient 10 single colonies were isolated. Characterization of each of these 10 single colonies by DNA fingerprinting as well as genotyping of several important genetic markers viz. *cagA, vacA, iceA*, *vapD*, *cag* PAI empty site, IS605, RFLP and two other genetic segments within *cag* PAI revealed that all of the 30 patients were infected with more than one strain and sometimes strains with 5 to 6 types of genetic variants. Analyses of certain genetic loci showed the microdiversity among the colonies from single patient, which may be due to the recombination events during long-term carriage of the pathogen. These results suggest that most of the patients have acquired *H. pylori* due to repeated exposure to this pathogen with different genetic make-up, which may increase the possibility of super infections. Genetic exchanges between these unrelated *H. pylori* strains may support certain *H. pylori* variant to grow better in a given host than the parental strain and thereby increasing the possibility for the severity of the infection.

## Introduction

Colonization in the stomach by the bacterium *Helicobacter pylori* is very common in humans, and the asymptomatic infection may lead to unwanted outcomes including peptic ulcers and gastric cancer. The infection frequencies vary from 20–30% in economically developed regions to 70–90% in developing regions like India. This infection often begins at the early stage of life and continues for many years. *H. pylori* has a remarkable ability to establish infections in human stomachs lasting for decades and is the major cause of peptic ulcer, gastritis and a risk factor for gastric cancer [1]–[3]. Interestingly, it is unclear why only about 10% of the infected individuals suffer from gastro-duodenal disease.

Transmission occurs mostly by faecal–oral routes and also through contaminated food, water and unclean hands [4]–[6]. *H. pylori* exhibits pronounced genetic diversity as evidenced by apparently unlimited number of unique strains that differ in genome size, gene order, genetic content, and allelic profiles [7]. Some of these essential genetic variations probably allow *H. pylori* to adapt to individual host conditions, and thus, contribute to the prolonged infection state.

Independent *H. pylori* strains can usually be distinguished from one another by methods such as arbitrarily primed polymerase chain reaction restriction fragment length polymorphism analysis, multilocus enzyme electrophoresis or the sequencing of one or a few representative genes [8]–[12]. A number of genetic determinants or phenotypical traits that distinguish individual *H. pylori* strains from one another and that relate to colonization or disease are also known. There is no non-human reservoir for this pathogen and this microaerophilic bacterium cannot survive for long periods outside the body. Therefore, the most probable place for genetic recombination is human gastric mucosa and it is possible that during the long-term colonization the *H. pylori* strains may undergo adaptive changes and eventually become significantly different from the ancestral genotype [13].

Indeed, *H. pylori* exhibits more frequent recombination events with heterologous strains than any other known bacterial species [14]. Microarray and nucleotide sequence analysis of strains isolated longitudinally from the same patient imply that this recombination is a continuous event [13], [15]. Studies from Europe and Western countries showed that almost all strains of *H. pylori* isolated from different sites in the stomach of individual patients show homogeneous DNA profiles. In contrast, Mexican and Chinese populations are infected with genetically heterogeneous strains with high infection rates. In India, the prevalence of *H. pylori* infection is high [16]–[18] and the chances of infection and re-infection of strains in single host is relatively more as compared to the Western populations. In addition, no investigation has been undertaken in India to determine the genetic types of different *H. pylori* strains from a single host. The aim of the present work was to examine the genetic diversity of *H. pylori* strains from Indian patients. We have assessed the extent of genetic diversity of multi *H. pylori* strains using DNA fingerprinting, genotyping and virulent gene profiling from same host. Our data strongly suggest that most of the patients exposed to repeated infection with the *H. pylori* containing different genetic character that increases the possibility of severe form of gastro-duodenal diseases.

## Results

### Studies on Colonization of Multiple *H. pylori* Strains in Same Host by RAPD Analysis

**Figure 1:**
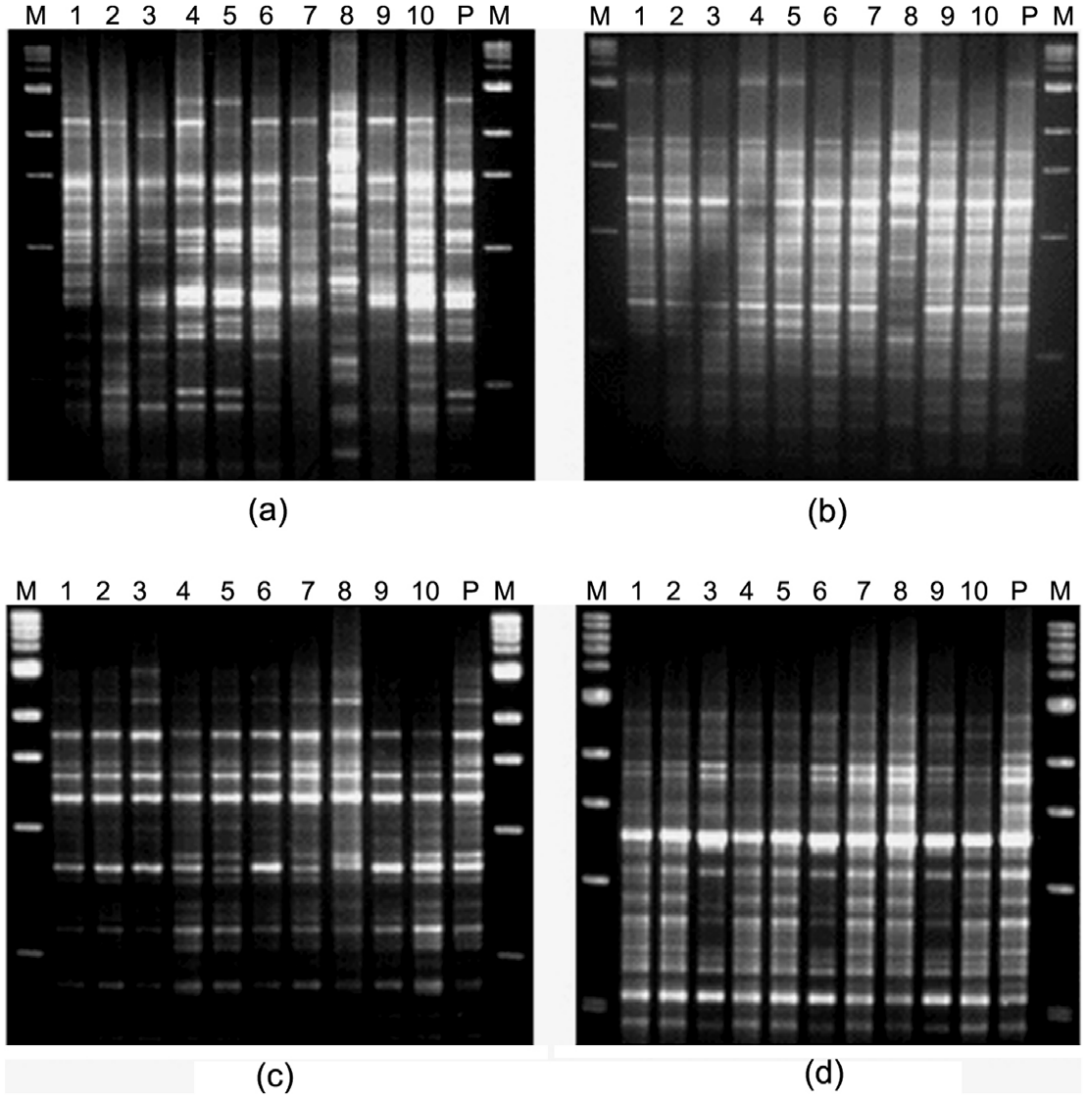
RAPD patterns obtained with primer 1281 and 1283 in two representative cases. M, 1 kb molecular marker (New England Biolabs); lanes 1–10, single colonies isolated from respective patients and “P” denotes pooled DNA sample (a) Multiple *H. pylori* colonies and pooled culture isolated from PG207 showed different RAPD patterns with primers 1281. (b) The same *H. pylori* colonies (PG207) gave different RAPD patterns with primer 1283 also. (c) Showing difference in RAPD patterns among single colonies obtained with primer 1281 for PG158 though, (d) almost identical RAPD patterns were obtained for these colonies with primer 1283.

Ten individual colonies and a pooled bacterial culture were isolated from each patient and typed by RAPD using random primers 1281, 1283 and PCR using primers targeted for alternate alleles of the same gene of *H. pylori*. The presence of multiple *H. pylori* strains in a single individual were detected in 25 patients by RAPD PCR using both primers 1281 and 1283. However, in 5 patients, multiple *H. pylori* strains were identified with any one of these primers (Figure 1).

### Genotype of Strains Isolated from Same Individuals

PCR primers designed in this study were to amplify several genetic loci among *H. pylori* strains. This strategy helped us to identify multiple *H. pylori* strains from a single host. All of the 30 patients had mixed infection ranging from 2 to 6 different strains (Table 1) in a single host with different combinations of genotypes.

**Table 1:** Over all analysis regarding the colonization of multiple *H. pylori* strains in same host in this study population.

	No. of Patients Infection With					
Disease Outcome	2 types ofcolonies	3 types ofcolonies	4 types ofcolonies	5 types ofcolonies	6 types ofcolonies	Mean no. ofcolonies
Duodenal Ulcer (n = 21)	9	7	5	0	0	2.8
Gastritis (n = 6)	3	1	1	1	0	3
Cancer (n = 3)	1	1	0	0	1	3.67

### Multiplex PCR for *vacA* and *cagA* Genotype

**Figure 2:**
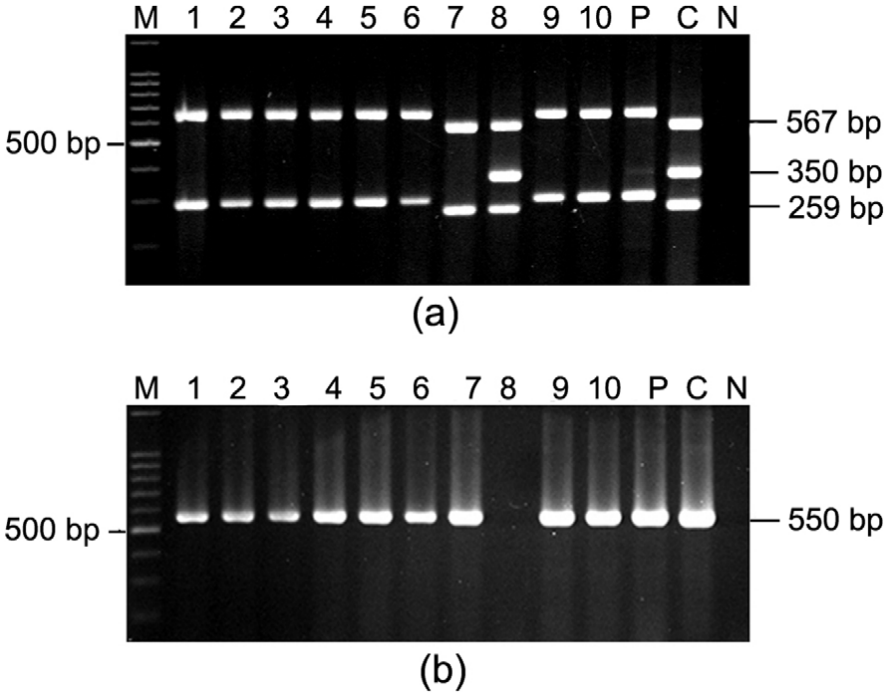
Multiplex PCR for *vacA* subtypes, *cagA* and *cag* PAI empty site for the absence of *cag* PAI. M, 100 bp marker (New England Biolabs); lanes 1–10, single colonies isolated from PG207; C, 26695 for the first set (a) and AM1 (*cag* PAI negative strain) for the second set (b); N, Negative control (*E. coli* DNA). (a) Multiplex PCR showed this particular patient was infected by at least three different strains. Lanes 1–6 and lanes 9–10 showed existence of s2m2*cagA* ^−^ strains, lane 7 showed existence of s1m1*cagA* ^−^ strain and lane 8 showed existence of s1m1*cagA* ^+^ strain. (b) All the single colonies, which failed to give amplicon for *cagA* gene, yielded ∼550 bp product for *cag* PAI empty site. The colony (Lane 8) that produced amplicon for *cagA* did not show any amplicon with primers for *cag* PAI empty site.

**Figure 3:**
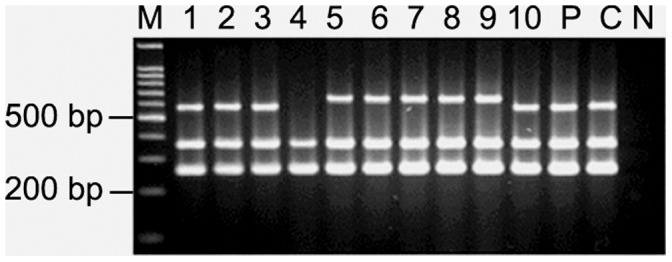
Multiplex PCR for *vacA* subtypes and *cagA* gene for PG218. M, 100 bp marker; lanes 1–10, single colonies isolated from PG218 and “P” denotes pooled DNA sample; C, Positive control (26695); N, Negative control (*E. coli* DNA). All the colonies isolated from this individual were *cagA* ^+^ and carried *vacA* s1 allele. Evidence of mixed infection was detected in the *vacA* middle region only. Lanes 1–3 and 10 yielded amplicon specific for m1, lanes 5–9 produced amplicon specific for m2 while in lane 4, no amplicon was obtained for *vacA* mid-region using this specific primer set.

Multiplex PCR was performed to identify differences if any in the *vacA* and *cagA* genes of *H. pylori* strains isolated from the same host. Among the 30 patients, 5 were identified with multiple *H. pylori* strains by having difference in *vacA* and *cagA* status. Two patients had both *cagA* ^+^ and *cagA* ^−^ strains. The colonies that were negative for *cagA* in multiplex PCR, yielded a 550 bp amplicon for *cag*-empty site. This showed that these strains truly lacked *cag* PAI and the failure to amplify *cagA* by multiplex PCR in these strains were not due to point mutation in *cagA* gene or partial deletion of *cag* PAI. In one of these two individuals (PG207), who had infected by both *cagA* ^+^ and *cagA* ^−^ *H. pylori* strains, also harbored at least 3 different strains including s1m1*cagA* ^+^, s1m1*cagA* ^−^ and s2m2*cagA* ^−^ genotypes. The gene *cagA* is usually associated with s1m1 allelic combination of *vacA* gene and in most of the strains that carry s2m2 allelic combination of *vacA* gene also lack *cagA*. One of the five strains isolated from this patient was found to carry a rarely described s1m1*cagA* ^−^ genotype, which was further confirmed by *cag*-empty site PCR (Figure 2). This finding support the hypothesis that strains differing in virulence potential can colonize in a host and support recombination events resulting emergence of unique variants [19]. The other individuals showed presence of both *cagA* ^+^ and *cagA* ^−^ strains either with s1m1*cagA* ^+^ or s2m2*cagA* ^−^ genotype. In 3 individuals, all the colonies carried *cagA* gene but with different *vacA* subtypes such as *vacA* s1 m1 or *vacA* s1m2 (Figure 3).

### Length Polymorphism at 3′ End of *cagA* Gene

**Figure 4:**
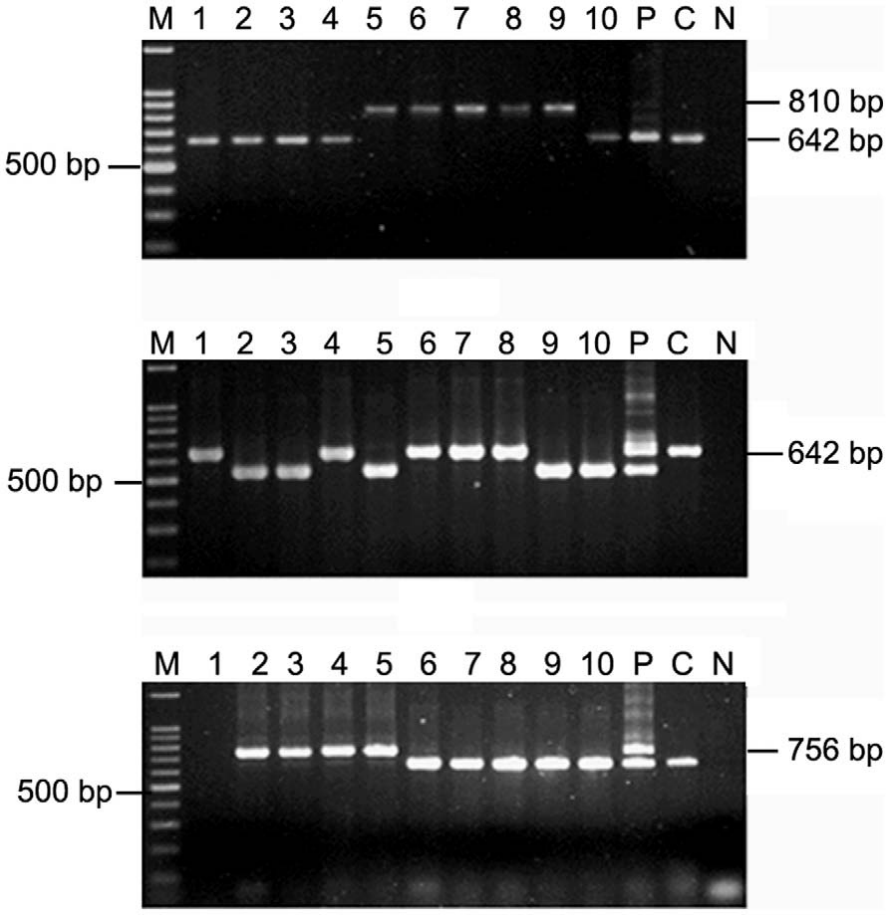
Variant *cagA* subtypes detected on the basis of PCR with primers CAG1 and CAG2 amplifying the 3′ end of the gene. M, 100 bp marker; lane 1–10, single colonies isolated from individual patient and “P” denotes pooled DNA sample; C, positive control for type A *cagA* (cagA types were named according to the types described by Yamaoka *et al.*, (1998); N, Negative control (*E. coli* DNA). (a) Mixed *H. pylori* populations were detected by obtaining amplicons for type A and type C in PG218. (b) For PG93, mixed *H. pylori* populations were detected by obtaining amplicons for type A and a shorter amplicon of ∼500 bp, which could not be typed by the methodology developed by Yamaoka *et al.*, (1998). (c) For PG144, mixed *H. p*ylori populations were detected by obtaining amplicons for type A and type B/D.

Polymorphism at the 3′ end of *cagA* gene was examined with colonies isolated from all the 30 patients to test the possibility that single individual might harbor *H. pylori* strains carrying two or more types of *cagA* gene. The CagA protein, carrying 5 or more phosphorylation sites were described to elicit significantly intense biological activity than CagA carrying only 3 or less phosphorylation sites. To identify such polymorphism, PCR with primers CAG1 and CAG2 were used to identify the type C that should encode 5 phosphorylation sites from the less number of phosphorylation site encoding type A, B and D. With this strategy, multiple *H. pylori* strains carrying different *cagA* types were detected in 10 of 30 individuals. Five individuals were infected with *H. pylori* strains carrying type A and type B/D *cagA* gene while one carried type A and type C *cagA* gene. In one individual, some of the colonies have type A *cagA* while the other colonies yielded shorter amplicon than type A. For other 3 individuals the *cagA* gene in some of the *H. pylori* colonies has been amplified and typed by PCR using CAG1 and CAG2 primers while the *cagA* gene in rest of the colonies could not be typed by this strategy (Figure 4).

### *iceA1* and *iceA2*

**Figure 5:**
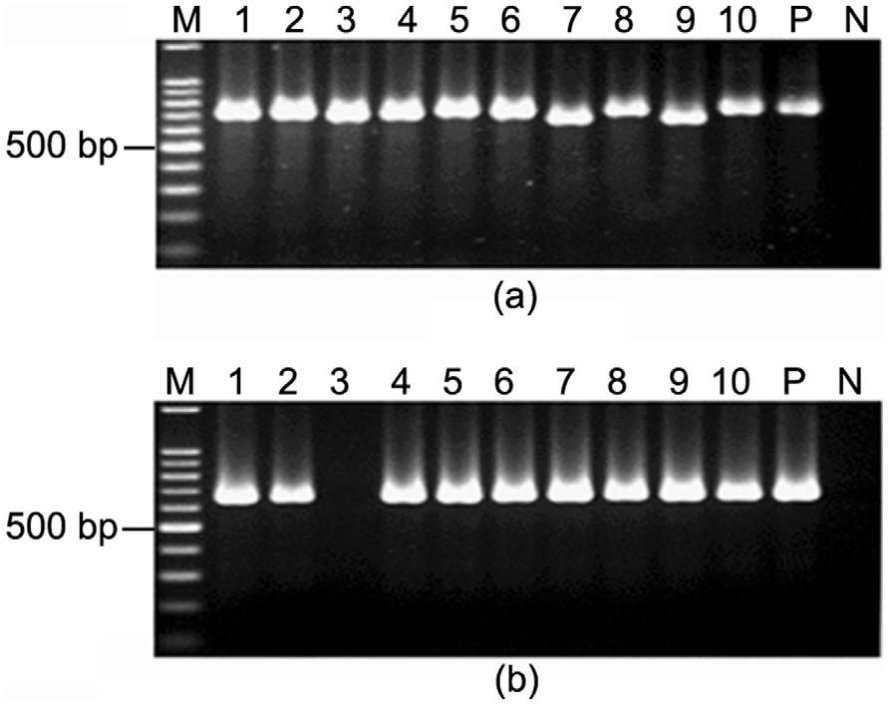
Mixed infections detected on the basis of *iceA* genetic locus. M, 100 bp marker; lanes 1–10, single colonies isolated from the patients, “P” denotes pooled DNA sample and N, Negative control (*E. coli* DNA). (a) PG98 showed evidence of mixed infections on the basis of obtaining amplicons of different sizes with primers specific for *iceA2*. (b) PG156 showed evidence of mixed infections on the basis of obtaining amplicons specific for *iceA2* and *iceA* negative genotype.

**Figure 6:**
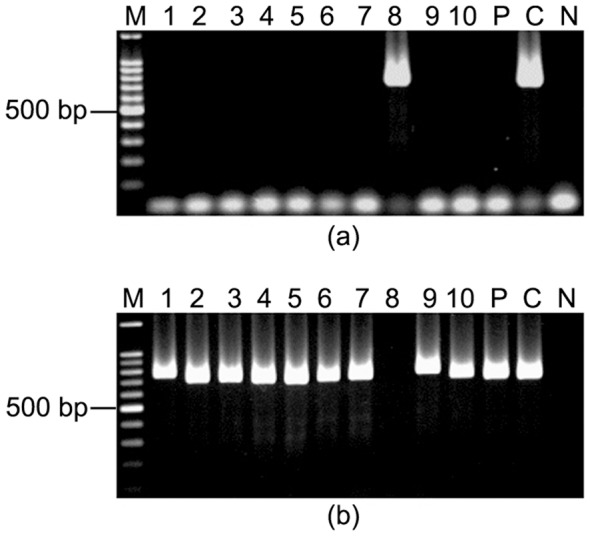
PG207 showed evidence of mixed infections on the basis of obtaining either *iceA1* or *iceA2* alleles. M, 100 bp marker; lanes 1–10, single colonies isolated from PG207; C, positive control; N, Negative control (*E. coli* DNA). (a) All the single colonies were negative for *iceA1* except for lane 8. (b) All these single colonies were positive for *iceA2* except for lane 8. Three different amplicon sizes were obtained in this *iceA2* PCR (lanes 1, 9; lanes 2, 3, 6, 7, 10 and lanes 4, 5).

In 7 of 30 individuals, colonization of multiple *H. pylori* strains were detected by PCR amplification based on *iceA* genetic locus. One of these 7 individuals was identified with *H. pylori* strains carrying *iceA1* and *iceA2.* Four of these 7 patients were infected with multiple *H. pylori* strains carrying *iceA2* amplicons of different sizes, while in 2 cases, mixed infections were detected by obtaining amplicon for *iceA2* and obtaining *iceA* negative genotypes (Figure 5 and 6).

### Genes within the *cag* PAI and IS*605* Insertion Sequence

**Figure 7:**
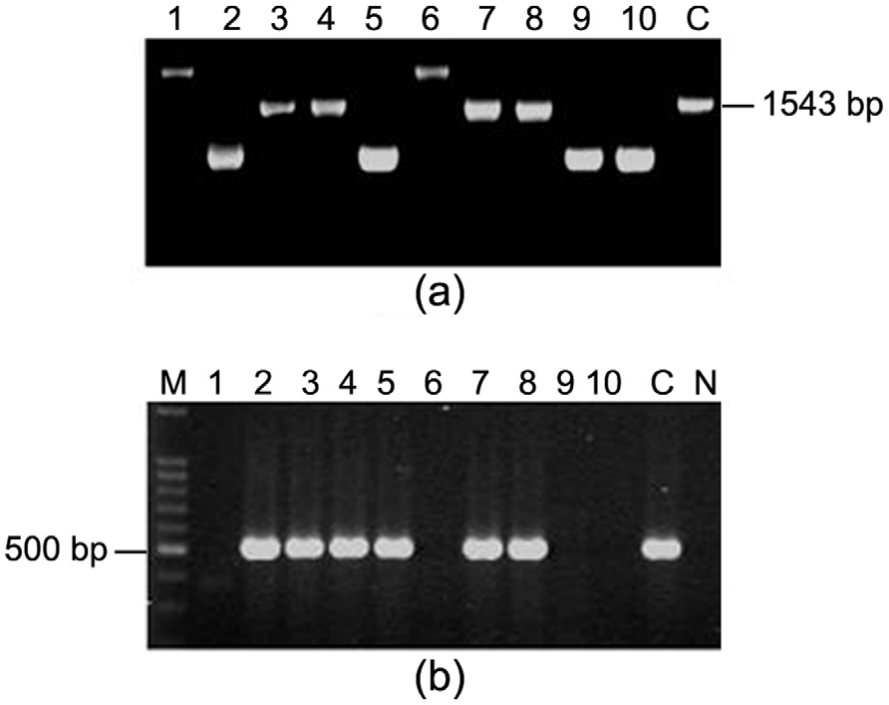
Multiple strain colonization detected on the basis of HP0527 gene in *cag* PAI. Lanes 1 to 10, single colonies isolated from the patient; C, Control strain 26695. (a) Three types of colonies were identified in PG93. Lane 1 and 6 gave a higher amplicon than that of 26695; lanes 3, 4, 7 and 8 yielded same amplicon while lanes 2, 5, 9, 10 produced lower amplicon than that of 26695. (b) Mixed infections detected on the basis of *vapD* genetic locus PCR. M, 100 bp marker; lanes 1–10, single colonies isolated from PG137; 11, positive control (PCR225); 12, Negative control (*E. coli* DNA). All the colonies are positive for *vapD* except colony numbers 1, 6, 9 and 10.

The genes within the *cag* PAI are highly diverse. In 9 of the 30 patients analyzed, several colonies failed to yield any amplicons for HP0527 gene in the *cag* PAI. In two patients (PG93 and PG144), three types of colonies were identified. In one type, colonies were failed to give any amplicon while the other two gave a higher and a lower amplicons than the expected size of the genetic locus of the strain 26695 (Figure 7a). This locus is one of the several *cag* PAI genes that codes for type IV secretion system needed for the transfer of variety of multi-molecular complexes across the bacterial membrane to the extracellular space or into other cells. Two types of strains, one gave positive amplicon and the other was negative with primer PAI 3S and PAI 3AS designed respectively between HP0522 and HP0523 of 26695, were found in 2 of 30 patients screened. Among the 30 patients studied, one had mixed infection with IS*605* positive as well as negative strains.

### VapD Chromosomal Region

The gene *vapD* is a 4.2-kb region downstream from *vacA* and contains an open reading frame (ORF) closely related to the virulence-associated protein D (*vapD*) gene of *Dichelobacter nodosus* [20]. A set of primers (vapD-F and vapD-R) designed from the conserved region of *vapD* gene was used to understand the presence or absence of this genetic locus. In this study, 15 patients showed mixed infections with *vapD* positive as well as *vapD* negative *H. pylori* strains (Figure 7b).

### PCR based RFLP

**Figure 8:**
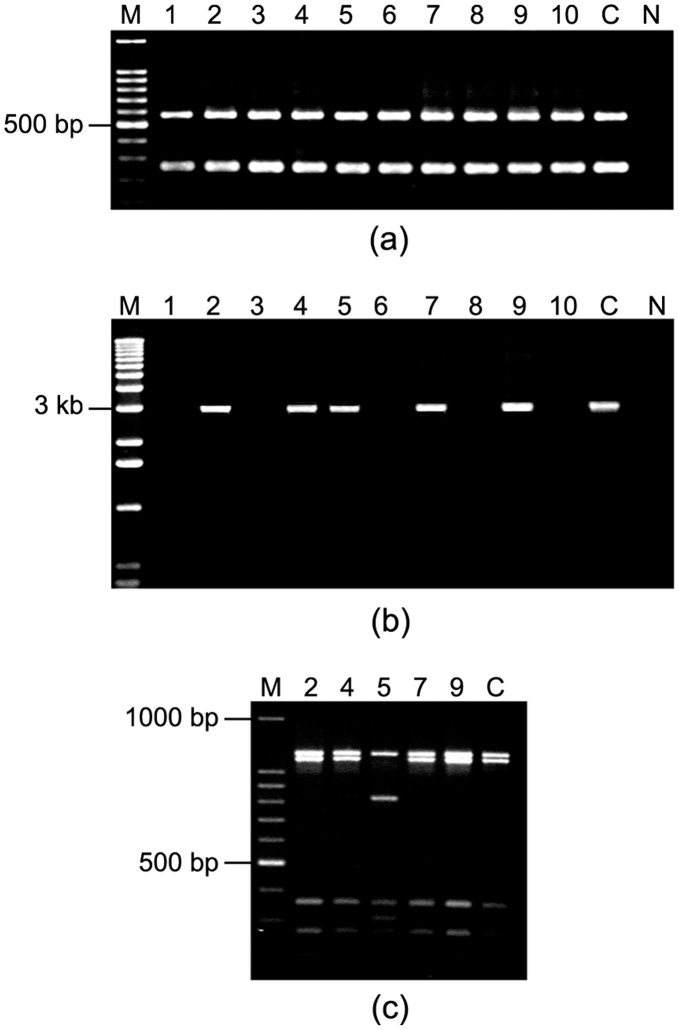
Analysis of *vacA* alleles by PCR and also the RFLP analysis of ∼ 3 kb **fragment of** ***vacA*** **.** M, marker; lanes 1 to 10; single colonies isolated from PG142; C, Positive control (26695); N, Negative control (*E. coli* DNA). (A) Amplification of *vacA* s1 and m1 alleles, Lane M, 100 bp marker; all the colonies were positive for *vacA* s1m1. (B) Amplification of the *vacA* region with primer vas1F and vas GR. Lane M; 1 kb marker (Gibco BRL), Lanes 1, 3, 6, 8 and 10 failed to amplify. (C) Restriction fragment length polymorphism (RFLP) analysis of ∼3 kb fragment of *vacA* region using *HaeIII* restriction enzyme depicted microdiversity among the isolates as lane 5 showed a different digestion pattern than the rest of the colonies. Lane M, 100 bp marker.

In the multiplex PCR, all the colonies from PG142 was *vacA* s1 m1 positive (Figure 8a). However, primers vas1F and vas GR (designed from *vacA*) successfully amplified ∼3 kb product representing an internal portion of *vacA* gene in 6 out of 10 colonies in one of the patient (Figure 8b). We were unable to obtain a PCR amplification product for the 4 other colonies of this patient (PG142) (Figure 8b). From the restriction enzyme digestion of these PCR products non-identical RFLP patterns was obtained (Figure 8c). Similar type of results was obtained in 8 other patients that showed mixed infection.

### Combination of RAPD and Genotype Data

**Figure 9:**
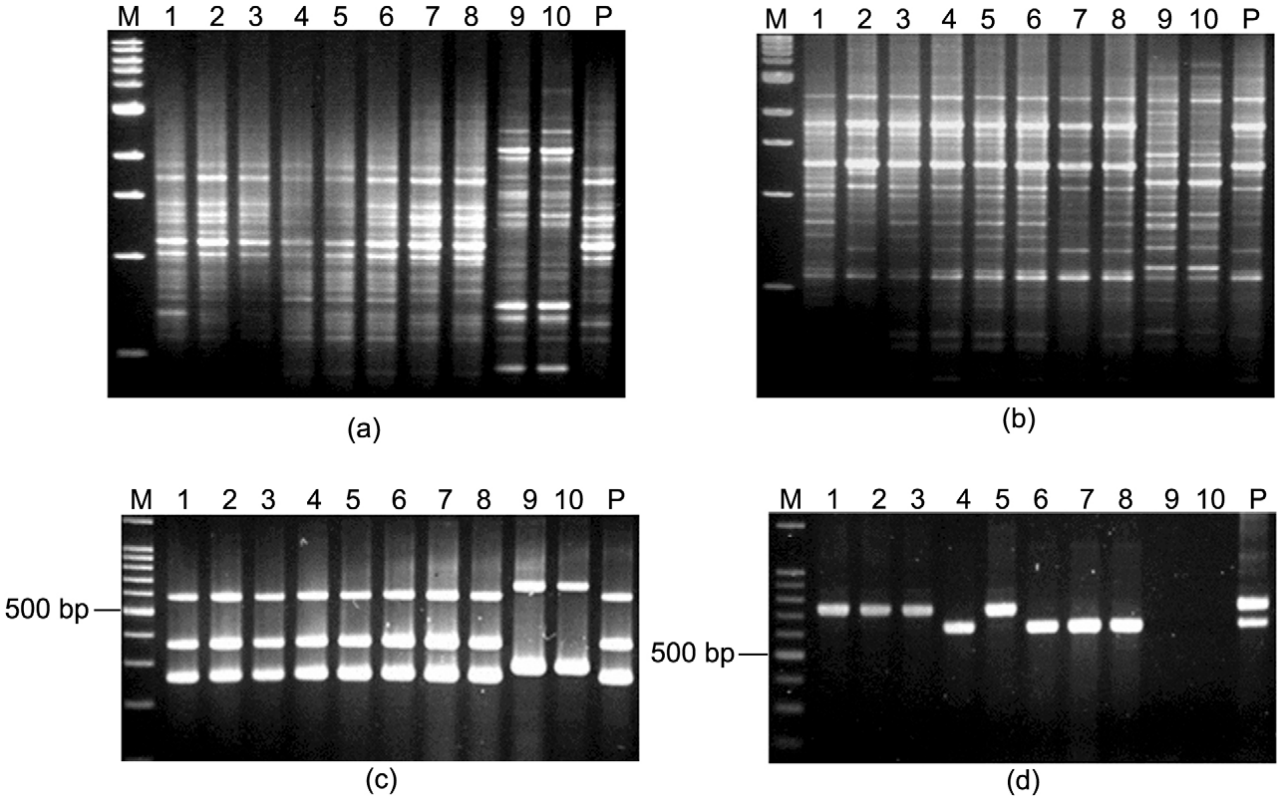
Combination of genotype and RAPD analysis for PG157 indicated multiple infections and microdiversity in a single host. M, 100 bp marker; lanes 1–10, single colonies isolated from PG157; P, pooled DNA. (a) RAPD patterns using primer 1281 showed two distinct patterns (lanes 1–8 and lanes 9–10) (b) RAPD patterns using primer 1283 also yielded two distinct patterns (lanes 1–8 and lanes 9–10) (c) Multiplex PCR for *vacA* alleles and *cagA* showed existence of s1m1*cagA* ^+^ strains in lanes 1–8 and s2m2*cagA* ^−^ strains in lanes 9–10. (d) Variant *cagA* subtypes detected on the basis of PCR for 3′ end of *cagA* using primers CAG1 and CAG2. This PCR assay showed existence of type A strains in lanes 4, 6–8 and existence of type B/D strains in lanes 1–3 and 5. Lanes 9–10, which were detected as *cagA* ^−^, did not produce any amplicon and the pooled sample yielded amplicons for type A and type B/D strains.

The genotype data obtained from multiplex PCR for *vacA* and *cagA*, 3′ end of *cagA*, *iceA*, IS*605*, IS*606*, HP0522, HP0523, HP0527, *vapD* and *vacA* genes using primers vas1F and vasGR, genetic locus analysis for all the single colony isolate from single individuals and their RAPD patterns were collectively analyzed. Strains isolated from same individuals, with or without *cag* PAI showed distinct variations in the RAPD patterns (Figure 9a and 9b). Relatively lesser variations in RAPD patterns were obtained among strains, which differed in other genetic loci. For example, PG157 was infected by at least 3 types of strains- (i) s1m1*cagA* ^+^ (Type A) (ii) s1m1*cagA* ^+^ (Type C) and (iii) s2m2*cagA* ^−^ (Figure 9c). Strains s1m1*cagA* ^+^ (Type A) and s1m1*cagA* ^+^ (Type C) showed very little differences, if at all, in RAPD patterns whereas s2m2*cagA* ^−^ strains showed different RAPD patterns with both primer 1281 and 1283 (Figure 9a and 9b).

### Microdiversity among the Isolates in a Single Host

**Figure 10:**
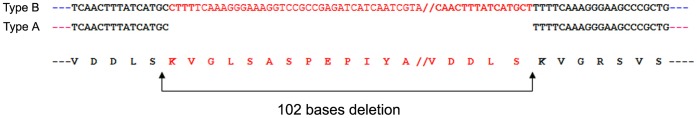
Sequencing analysis of both Type A and Type B *cagA* 3′end amplicon of PG157 showed the deletion of 102-bp in type A strains, which contained one “EPIYA” motif.

During analysis of 10 single *H. pylori* colonies from PG157, two different RAPD patterns were obtained; colonies 1–8 showed one pattern and colonies 9 and 10 showed another pattern (Figure 9a and 9b). In the multiplex PCR, colonies 1 to 8 were *cagA^+^*, *vacA* s1m1 while colonies 9 and 10 were negative for *cagA* ^−^ but positive for *vacA* s2m2 (Figure 9c). This result indicated deletion of a whole 40 kb fragment i.e. *cag* PAI in PG157 9B and 10B with difference in the *vacA* allele. Further analysis of the 8 colonies at the 3′end of the *cagA* with CAG1 and CAG2 primers revealed that some colonies gave an amplicon size of 642 bp (*cagA* Type A) while others gave 756 bp (*cagA* TypeB/D) (Figure 9d). Sequencing analysis showed that *cagA* Type A has three EPIYA (TPMs) motifs while *cagA* Type B/D has 4 EPIYA motifs. There was a deletion of 34 amino acids in *cagA* Type A, and it had made this difference in the number of EPIYA motifs (Figure 10). Further analysis to find whether the two strains have the same origin, we performed sequencing analysis of the two housekeeping genes, *ureB* and *recA*. These two genes showed 100% homology in both the strains. Co-existence of variants of the same strain with different *cagA* genotypes may reflect the physiological differences among gastric regions of a given host that would select for derivatives that adapted bettering other available locations [21].

## Discussion

Horizontal gene transfer is the major contributing factor in the development of bacterial diversity [22], [23]. *H. pylori* exhibits remarkable genetic diversity, as evidenced by variation in gene order, differences in genetic content, and mosaic nature of functional genes [8], [11], [12], [24]–[29] Natural competence in this organism was discovered within 8 years of its first successful culture and later recombination in this organism was estimated to be higher as compared to other bacterial species. Diversity among *H. pylori* isolates can be understood in a population genetic context, based on a proposed human diversity in traits that affects *H. pylori* growth and a tendency of established infections to persist for years or decades [30]. *H. pylori* has panmictic population structure, DNA-fingerprint of two unrelated strains, isolated from any two unrelated individuals usually show non-identical patterns, which is indicative of frequent genetic exchange as well as co-evolution of this persistent gastric colonizer with its host. Therefore, colonization of the multiple-strains in single host is a prerequisite for this microaerophillic species for horizontal gene transfer as well as adaptation to a niche, where only few other microorganisms can colonize. Colonization of individuals with more than a single strain appears to be rare among Europeans and North Americans. Taylor *et al.* (1995), using RAPD, found more than one strains in only three out of 15 patients screened [5]. Marshall (1996), using oligofingerprinting, found two strains in only one out of 19 patients screened [31]. Shortridge *et al*. (1997) using PCR RFLP of *ure C* genes, found multiple strains in only two out of 81 patients examined [32].

Numerous studies indicate that mixed *H. pylori* infections may be quite common. Yakoob *et al.* (2000) showed that two different *H. pylori* strains coexist in an infected individual and may not be uniformly distributed among biopsy sites [33]. Heterogeneity of protein profile of *H. pylori* isolated from individual patients was shown by Kitamoto *et al.* (1998) [34]. Heterogenic nature of *H. pylori* strains among infected couples has also been reported from Taiwan [35]. Attempts to assess this microdiversity in single host in western countries have resulted in different outcomes. Although, *H. pylori* infection is extremely common in India virtually no study has been conducted in India to address the level of multiple infection in a single host. We undertook this study to assess the microdiversity of *H. pylori* strains in a single gastric niche, by RAPD-fingerprinting, PCR based RFLP and PCR amplification of alternate alleles for multiple colonies and pooled cultures isolated from 30 individuals.

All the patients included in this study carried multiple *H. pylori* strains in their gastric mucosa, as evidenced in the RAPD-fingerprint analysis using two random primers, 1281 and 1283. Differences seen with RAPD patterns of *H. pylori* strains were very less but with variation in a single genetic locus. For example, one patient carried multiple *H. pylori*-strains with *cagA* subtypes but showed very little difference in RAPD patterns, confirming a common genetic back borne. Similar findings were also obtained in strains isolated from single hosts, carrying *vacA* m1 or *vacA* m2 allelic types and *iceA1* or *iceA2* alleles. However, strains with *cag* PAI positive or *cag* PAI negative genotypes, though isolated from same individual gave different RAPD-fingerprint. This indicates, *cag* PAI positive strains are phylogenetically distinct from *cag* PAI negative strains colonized in a single gastric niche or presence or absence of ∼40 kb PAI could be detected with precision by two random primers used in our study than strains with differences in *cag A* 3′ end or in other genetic loci tested.

Isolation of multiple colonies and DNA-fingerprint analysis was previously tested by other workers and the results obtained by them are variable. Miehlke *et al*. (1999), for example, reported earlier that in Columbian cancer patients, usually a single predominant strain exists [36]. But in our study, all 3 cancer patients were colonized with multiple strains, as evidenced both by DNA fingerprinting and genotyping. The possible explanation for this difference could be that the Columbian patients rarely get exposed to multiple *H. pylori* strains or due to the use of lesser discriminatory REP-PCR. For *H. pylori*, RAPD is the best known technique for discriminating two strains than other typing techniques [37]. However, use of single primer is often not sufficient in the RAPD and two primers are generally required to confirm the genetic difference among two strains. Results obtained in our study are in agreement with Israel *et al*. (2001) who showed evidence of microdiversity in terms of close relatedness in DNA-fingerprints of strains isolated from the same individual [13].

To our knowledge this study was the first attempt to demonstrate existence of multiple *H. pylori* strains with different genetic polymorphisms in a single host in Indian subcontinent. Genetic exchanges among mixed bacterial population may generate a more competitive strain to adapt to a particular host and thereby propagating a more virulent strain. In India, prevalence of *H. pylori* infection is much higher as compared to the most western countries and almost all infected cases were found to carry multiple *H. pylori* strains. This heterogeneity of *H. pylori* population is clinically very important as the exiting practice of characterization of single isolated from infected individuals may oversight the appropriate target of virulence gene or its alleles. In addition, due importance should be given to the patchy distribution of *H. pylori* throughout the gastric mucosa, as different genotypes may predominate at different sites. Our finding strengthens the view of including multiple colonies from a single host either from single or different sites in the case of multi ulceration.

## Methods

### Ethics Statement

The Ethical Committee of the Institute of Post Graduate Medical Education and Research (IPGMER), Kolkata and National Institute of Cholera and Enteric Diseases (NICED), Kolkata, India approved this study.

### Collection of Biopsies

Biopsies were obtained from antrum and corpus of the stomach from 30 patients admitted in the IPGMER, Kolkata with gastro-duodenal diseases including peptic ulcer disease (n = 21), gastritis (n = 6), and gastric adenocarcinoma (n = 3). All these patients underwent a non-sedated upper gastrointestinal endoscopy (GIF XQ 30, Olympus Optical Company, Japan) under topical lignocaine anesthesia. From each patient, 10 single colonies and a pooled bacterial culture were isolated and characterized as described below.

### Culture of *H. pylori*

Gastric biopsy specimens were transported in 600 µl of Brucella broth (Difco, Detroit, Mich.) containing 25% of glycerol in cold condition (4°C) for culture at the NICED within 1 hour of collection. In the laboratory, the specimens were vortexed for 2 min and 200 µl of the mixture was plated on petri plates containing brain heart infusion (BHI) agar (Difco supplemented with 7% horse blood, 0.4% IsoVitaleX, amphotericin B (8 µg/ml) (Sigma, St. Louis), trimethoprim (5 µg/ml) (Sigma), and vancomycin (Sigma) (6 µg/ml). The plates were incubated at 37°C in an atmosphere of 5% O_2_, 10% CO_2_ and 85% N_2_ for 3–6 days in a double gas incubator (Hera cell 150i, Thermo fisher scientific, Waltham, Ma, USA). *H. pylori* colonies, which appeared as translucent water droplets, were identified based on their typical morphology, as well as biochemical tests like urease, oxidase and catalase tests. The *H. pylori* strains were preserved in sterile BHI broth containing 20% glycerol at −70^o^ C.

### Randomly Amplified Polymorphic DNA-PCR (RAPD-PCR)

RAPD-PCR reaction was carried out in 25 µl (l volume containing 25 pmol of primer 1281 or primer 1283, 0.25mM of each dNTP, 1.5 U of *Taq* DNA polymerase, and 4 mM of MgCl_2_. After initial denaturation at 94^o^ C, the products were amplified for 45 cycles at 94^o^ C for 1 min, 36^o^ C for 1 min, 72^o^ C for 2 min and finally extended for 10 minutes at 72^o^ C in a Perkin-Elmer 9700 thermocycler, as described previously [38]. The products were resolved in 2% agarose gel and stained with 0.5 µg/ml ethidium bromide solution. A 1 kb DNA ladder (New England Biolab, Ipswich, MA, USA) was used as a molecular weight marker.

### Characterization of *H. pylori* Strains by PCR

*H. pylori* genomic DNA was extracted by CTAB (hexadecyltrimethyl ammonium bromide) method [39] from 24 hr grown confluent bacterial culture on brain heart infusion agar (BHIA; Difco). Specific PCR was carried out in 20 µl volume containing 10 ng of bacterial genomic DNA, 20 p mole of each primers, 0.25 mM of each dNTPs (Takara, Shuzo, Japan), 1 U of Taq DNA polymerase (Takara) in standard PCR buffer (Takara) containing 1.5 mM MgCl_2_. PCR products were amplified for 35 cycles at 94°C for 1 min, 55°C for 1 min and 72°C for 1 min (1 min/kb) unless otherwise stated. The primers are listed in Table 2. PCR products were purified with the QIA quick gel extraction kit (Qiagen Corporation, Chatsworth, CA) according to the manufacturer’s instruction and were directly sequenced using the Big Dye terminator cycle sequencing kit (Perkin-Elmer, Applied Biosystems, Foster City, CA.) on an automated DNA sequencer (ABI Prism 310). DNA sequence editing and analysis were performed with programs in the GCG package (Genetics Computer Group, Madison, Wis).

**Table 2:** Nucleotides used in this study.

Region	Primer	Nucleotide sequence	Reference
*vacA* s1/s2	VA1-F	5′-ATG GAA ATA CAA CAA ACA CAC	[40]
	VA1-R	5′-CTG CTT GAA TGC GCC AAA C	
*vacA* m1/m2	VAG-F	5′- CAATCTGTCCAATCAAGCGAG	[40]
	VAG-R	5′- GCGTCAAAATAATTCCAAGG	
*cagA* (5′ end)	cag5c-F	5′-GTTGATAACGCTGTCGCTTCA	[40]
	cag3c-R	5′- GGGTTGTATGATATTTTCCATAA	
*cag* PAI Empty site	Lunil 1	5′-ACA TTT TGG CTA AAT AAA CGC TG	[40]
	R5280	5′-CCA ACG TGC GTA AAA GGG AAT TAG	
*cagA* (3′ end)	CAG1	5′ –ACCCTAGTCGGTAATGGGTTA	[41]
	CAG2	5′ –GTAATTGTCTAGTTTCGC	
*iceA*1	*iceA*1F	5′-TAT TTC TGG AAC TTG CGC AAC CTG AT	[46]
	M.Hpy1R	5′-GGC CTA CAA CCG CAT GGA TAT	
*iceA*2	cys SF	5′-CGG CTG TAG GCA CTA AAG CTA	[46]
	*iceA*2	5′-TCA ATC CTA TGT GAA ACA ATG ATC GTT	
*IS605*	ORF 18F	5′-CGC CTT GAT CGT TTC AGG ATT AGC	[46]
	ORF 18R	5′-CAA CCA ACC GAA GCA AGC ATA ATC	
*IS606*	FB1	5′-GAA TGT AAT TCT ACC TAA TCC TCC ATT C	[46]
	RB8	5′-GAG AAA CCT TGA TTG TTC CAT G	
*HP0527*	PAI 14S	5′-CAA TCT AGC GCC ACT TGA AC	[47]
	PAI 15AS	5′-CTA TGG TGA ATT GGA GCG TGT G	
*HP0 522-523*	PAI 3 S	5′-CAT CAC AGG CTC ATT AGA G	[47]
	PAI 3 AS	5′-CTG TTG TTC AAC CCT AGA GAG	
*vacA*	vas 1F	5′-AGC CGA TAG CAT CAG AGA AGA GC	This study
	vas GR	5′-CCC GCA TCA TGG CTA TCA ATC AAT	
*vapD*	vapD F	5′-AGA GAT GCG GTG AAT GG	[42]
	vapD R	5′-AAG CGT TAT GAG TGG TGT G	
RAPD	1281	5′-AAC GCG CAA C	[38]
	1283	5′-GCG ATC CCC A	

### Characterization of Strains by Multiplex PCR

Multiplex PCR for the characterization of *vacA* s1, *vacA* s2, *vacA* m1, *vacA* m2 and *cagA* was carried out in 25 µl volume containing 2.5 pmol of primers VAG-F and VAG-R, 25 pmol of primers VA1-F and VA1-R, 10 pmol of primers cag5c-F and cag3c-R, 0.25 mM of each deoxynucleoside triphosphate (Takara), 0.9 U of Taq DNA polymerase (Genei, Bangalore, India), and 1.5 mM of MgCl_2_ in standard PCR buffer (Takara). Products were amplified under the following conditions: 3 min at 94°C for initial denaturation followed by 35 cycles of 1 min at 94°C, 1 min at 55°C, and 1 min at 72°C, in a Perkin-Elmer 9700 thermal cycle [40].

### 3′ End of *cag A* and *VapD* Chromosomal Region

All the strains, which were positive for *cagA*, were used to amplify 3′ end repeat region of *cagA* gene with primers CAG1 and CAG2 [41]. *vapD* is a 4.2-kb region downstream from *vacA* and contains an open reading frame (ORF) closely related to the virulence-associated protein D (*vapD*) gene of *Dichelobacter nodosus* [20]. The *vapD* chromosomal region was amplified using primers vapD-F and vapD-R [42].

### PCR-RFLP Analysis

PCR- amplified *vacA* segments were digested with *Hae*III (Bangalore Genei) for 3 hrs at 37°C in the appropriate buffer recommended by the supplier and the digested DNA were analyzed on ethidium bromide stained 2% agarose gel [34], [43], [44]. PCR amplification of the *vacA* gene fragment was done using primers vas1F and vasGR [45].
